# Does Camouflaging Cause Reduced Quality of Life? A Co-Twin Control Study

**DOI:** 10.1007/s10803-024-06583-0

**Published:** 2024-10-12

**Authors:** Karl Lundin Remnélius, Janina Neufeld, Johan Isaksson, Sven Bölte

**Affiliations:** 1 Department of Women’s and Children’s Health, Karolinska Institutet, Center of Neurodevelopmental Disorders at Karolinska Institutet (KIND), Stockholm, Sweden; 2 Swedish Collegium for Advanced Study (SCAS), Uppsala, Sweden; 3 Child and Adolescent Psychiatry Unit, Department of Medical Sciences, Uppsala University, Uppsala, Sweden; 4 Region Stockholm, Child and Adolescent Psychiatry, Stockholm Health Care Services, Stockholm, Sweden; 5 Curtin Autism Research Group, Curtin University, Curtin School of Allied Health, Perth, Australia

**Keywords:** Quality of life, Camouflaging, Sex, Gender, Autism, Twin design

## Abstract

Camouflaging has been proposed to have a detrimental effect on quality of life, yet previous research has not accounted sufficiently for potential confounding by genetic and shared environmental factors. The current study utilized a co-twin control design providing stringent control for a range of confounders to investigate the hypothesis that camouflaging autistic traits has a negative impact on quality of life. The sample included 140 individual twins from 42 monozygotic (MZ) and 28 dizygotic (DZ) twin-pairs, enriched for participants with neurodevelopmental conditions including 22 autistic participants. All twins provided self-reports of camouflaging and quality of life. Autistic participants and specifically autistic females displayed increased camouflaging behaviors compared to non-autistic participants. Across the sample, higher levels of camouflaging were associated with reduced quality of life, surviving adjustment for confounding effects of autistic traits, ADHD, sex, and age. Within DZ- as well as MZ-pairs, which provide the highest level of control for unmeasured confounders, twins who camouflaged more reported lower quality of life compared to their co-twins, consistent with a causal influence of camouflaging on quality of life. Our results strengthen previous claims purporting camouflaging behaviors as a risk factor for reduced quality of life.

## Introduction

The past decades have seen a shift in the focus of healthcare, from primarily aiming to eliminate disease or reduce symptoms to later years’ inclusion of the subjective appraisal of health-related quality of life (QoL) as a key aspect of health (World Health Organization, 1998). QoL is defined by the World Health Organization (WHO) as the “individuals’ perceptions of their position in life in the context of the culture and value systems in which they live and in relation to their goals, expectations, standards and concerns”, connecting the construct to the WHO conceptualization of health as defined not only by absence of disease but also encompassing aspects of physical, mental, and social wellbeing (WHO, 1998, p. 4). As highlighted by the definition, QoL emphasizes subjective appraisal and is generally understood as a multifaceted concept encompassing physical (e.g., energy in everyday life), psychological (e.g., self-esteem), social (e.g., satisfaction with personal relationships), and environmental domains (including satisfaction with living conditions, physical safety) (Power et al., 2003; The Whoqol Group, 1998).

A multitude of factors have been linked to reduced QoL, including the presence of mental health conditions, chronic medical conditions, and neurodevelopmental conditions such as autism (Cook & Harman, 2008; Jonsson et al., 2017; Olatunji et al., 2007; van Heijst & Geurts, 2015). Genetic factors also play a role, where a meta-analysis of twin studies found QoL to be moderately heritable (Bartels, 2015). Genetic factors might also partly explain phenotypic correlations between proposed risk factors and QoL. For example, the link between psychopathology and QoL is suggested to be explained in part by common genetic factors influencing both constructs (Bartels et al., 2013).

In addition to previously reported potential risk factors, research on social coping strategies to mask or compensate for autistic traits has proposed that extensive use of such *camouflaging* strategies might contribute to lower QoL (Hull et al., 2018). Originally characterized by autistic people, camouflaging is described as encompassing a range of behaviors including forcing oneself to make eye-contact, learning social behaviors from television or movies, and having prepared “scripts” for conversations (Hull et al., 2017). The use of such strategies has been described as motivated by both internal and external factors, including social motivation and the desire to develop relationships, but also to avoid being targeted or bullied, and be accepted by others (Cage & Troxell-Whitman, 2019; Hull et al., 2017). While reported by both males and females on the autism spectrum, use of camouflaging appears particularly extensive in autistic females (Bölte et al., 2023; Hull et al., 2019; Wood-Downie et al., 2020), potentially fueled by differences in sociocultural expectations on girls and boys (Kreiser & White, 2014).

When self-report measures are used, camouflaging behaviors are endorsed also by non-autistic individuals, but on average to a lesser degree compared to autistic people (Hull et al., 2018, 2019; Jedrzejewska & Dewey, 2022). These behaviors overlap with strategies described by autistic people, and may reflect attempts to act in accordance with social norms or ”not seem different”, by e.g., closely monitoring oneself, copying learned behaviors from others, along with experiences of ”performing” rather than being oneself in social interactions (Ai et al., 2023). Camouflaging among people who are not diagnosed with autism could reflect the distribution of autistic traits across the general population, which is supported by findings of correlations between autistic traits and camouflaging in autistic and non-autistic people (Hull et al., 2018; Lundin Remnélius & Bölte, 2023). Recent research also suggests that people with ADHD may be more prone to use camouflaging strategies than people without autism or ADHD diagnoses (van Der Putten et al., 2024). In addition, camouflaging may overlap with general human tendencies to attempt to influence others’ impressions of oneself, termed *impression management* (Ai et al., 2022; Goffman, 1959; Hull et al., 2018). Some aspects appear to differ between groups however, e.g., use of camouflaging increases during adolescence in both autistic and non-autistic groups but decline during adulthood in those without autism while often remaining at elevated levels among autistic adults (Lundin Remnélius & Bölte, 2023). The construct validity of camouflaging, including how autism-specific such strategies are, has sparked ongoing discussions (see Fombonne, 2020; Lai et al., 2021; Williams, 2022).

Research findings suggest that camouflaging autistic traits is linked to negative outcomes including reduced QoL in both autistic and non-autistic populations, which has been interpreted as detrimental consequences of camouflaging. Autistic and non-autistic adolescents report that camouflaging lead to experiences of being inauthentic, including not displaying one’s true self or not having “true” friendships (Bernardin et al., 2021b). Similarly, both autistic and non-autistic adults express that camouflaging autistic traits has negative effects on mental health, describing that these strategies can be stressful and taxing, and lead to self-criticism and rumination when the efforts have not been successful (Livingston et al., 2019). Consistent with these qualitative data, camouflaging shows associations with anxiety and depression, and is negatively associated with psychological wellbeing (often defined as a subdomain in QoL) in both autistic and non-autistic people (Bernardin et al., 2021a; Hull et al., 2018; Perry et al., 2022). In a previous study, we confirmed that camouflaging was negatively associated with self-reported QoL in a sample including autistic and non-autistic participants (Lundin Remnélius & Bölte, 2023). Similarly, a recent study found that camouflaging was linked to lower psychological QoL in people with an autism diagnosis, and to lower social QoL among males without a diagnosis of autism (Milner et al., 2022). In addition, a study investigating the link between camouflaging and lifetime suicidality among non-autistic young adults found that camouflaging showed an indirect association with lifetime suicidal thoughts and behaviors mediated by thwarted belongingness, i.e., feeling alone and disconnected from others (Cassidy et al., 2020). In summary, recent cross-sectional data indicate that extensive camouflaging of autistic traits is linked to negative outcomes including diminished QoL in both autistic and non-autistic populations.

### Counterfactual Reasoning and the Co-Twin Control Design

While previous research on camouflaging showed negative associations with QoL, a range of alternative explanations of the association have not been ruled out and no causal relationship has currently been established. Importantly, the association could be due to confounding factors influencing both factors rather than camouflaging having an impact on QoL. A potential source of confounding are genetic factors, which are known to commonly have pleiotropic effects were genes not only influence one specific trait but are involved in the predisposition of multiple traits (Bulik-Sullivan et al., 2015). As mentioned above, associations between QoL and other traits are reported to be largely explained by common genetic factors, i.e., that genes influencing other traits such as optimism or the risk of mental illness also influence subjective QoL (Bartels et al., 2013; Mosing et al., 2009).

Theoretically, a causal effect can be described as the difference in outcome if an individual is exposed to a risk factor compared to the outcome if the same individual had not been exposed (D’Onofrio et al., 2020). As both scenarios (the one that actually took place and the one that did not, the counterfactual) are not possible to observe directly, research methods are restricted to counterfactual inference models (Rutter, 2007). Randomization to different levels of exposure (e.g., to treatment or control group) is considered the gold-standard for making causal inferences, where the control group is thought to provide an average counterfactual to the treatment group as confounding variables are randomized to the groups (Shadish et al., 2002). While randomization can be unethical and is not feasible for many exposures of interest, observational studies using a *co-twin control design* can approximate a randomized experiment (McGue et al., 2010). In the co-twin control design, twin-pairs that are discordant for an exposure (here, one twin is camouflaging to a greater extent than the co-twin) can provide reasonable counterfactuals for one another – i.e., what would have been if the first twin camouflaged to a lesser degree (McGue et al., 2010). Comparisons within twin-pairs, assessing if the twin who reports increased camouflaging also reports lower QoL compared to their co-twin, adjust for a range of unmeasured familial confounders as factors shared within twin-pairs are held constant, including shared environment and genetic factors (D’Onofrio et al., 2020). Control for unmeasured familial confounding is not in itself sufficient for inferring causality (Scurrah & Hopper, 2019), but nevertheless allows for ruling out alternative non-causal explanations (D’Onofrio et al., 2020). Therefore, if the previously reported association can be found within monozygotic (MZ) twin-pairs which provides the highest level of control for unmeasured confounding, findings are consistent with a causal effect of camouflaging on QoL (McGue et al., 2010).

Given the hypothesis of a negative influence of camouflaging on QoL, further evaluation of the proposed causal effect is warranted, and of particular importance in populations where camouflaging is elevated such as among autistic people.

### Aims

The current study used a co-twin control design in a sample of MZ and dizygotic (DZ) twins enriched for autistic and other neurodivergent participants, allowing for control of unmeasured familial confounders which have not been considered in previous studies of presumptive negative effects of camouflaging. We set three specific aims for the study. First, to assess the relationship between autism and camouflaging and whether camouflaging levels are different among autistic females and males, as previously reported. Second, to investigate the relationship between camouflaging and QoL across the sample of twins while controlling for potential measured confounders including autistic traits and ADHD; and third, to test the association between camouflaging and QoL within twin-pairs, implicitly adjusting for everything shared within a twin-pair. We hypothesized a negative association between camouflaging and QoL, both across individuals and within DZ and MZ twin-pairs, consistent with a causal effect of camouflaging on QoL.

## Methods

### Participants

The sample included 140 individual twins, comprising 70 same sex twin-pairs (42 MZ and 28 DZ pairs), aged from 10 to 41 years. All twins participated in the Roots of Autism and ADHD Twin Study in Sweden (RATSS) (Bölte et al., 2014; Myers et al., 2021). Twenty-two participants fulfilled DSM-5 criteria for autism, 26 participants had ADHD, and 32 were diagnosed with at least one internalizing psychiatric condition, e.g., specific phobia, social anxiety disorder, major depression, or dysthymia. Note that participants can be included in more than one of the above diagnostic groups (e.g., participants can have diagnoses of both autism and ADHD). In the final sample, seven MZ twin pairs were discordant for autism diagnosis (two female and five male pairs), and three MZ pairs were concordant for autism (one female and two male pairs). In DZ twins, nine pairs were discordant for autism (four female and five male pairs), while no pairs were concordant for autism. See Table 1 for sample characteristics.

**Table 1:** Sample characteristics

	Non-autistic		Autistic	
	Males, *n* = 52	Females, *n* = 66	Males, *n* = 14	Females, *n* = 8
Age^1^, *M* (*SD*), Range	20.7 (8.2), 12–37	25.8 (7.5), 11–41	15.1 (7.3), 10–37	22.5 (6.8), 11–31
ADHD, *n*	9	7	6	4
Internalizing conditions, *n*	9	19	0	4
IQ, *M* (*SD*)	106.7 (14.0)	103.8 (11.5)	98.5 (22.8)	103.6 (24.2)
CAT-Q/SE total score, *M* (*SD*)	60.7 (17.9)	60.5 (25.6)	61.6 (20.6)	106.2 (15.4)
EUROHIS-QOL total score, *M* (*SD*)	30.6 (6.4)	28.7 (6.2)	32.3 (6.4)	24.1 (4.1)

^1^ Age when completing CAT-Q/SE and EUROHIS-QOL. CAT-Q/SE: Camouflaging Autistic Traits Questionnaire, Swedish version

All participants were verbal (i.e., used basic level of functional language as indicated by parent-report from the Autism Diagnostic Interview-Revised (ADI-R), or by educational level (for one pair, where both twins had completed university educations). Among the twins younger than 18 years at their visit in RATSS, parents reported that 90% were in education while information was not available from the remaining 10%. Among the twins that were 18 years or older at their visit, 13% had finished primary and lower secondary education, 85% had completed upper secondary education or higher, and for 1% information on education level was not available.

Twins are more likely than singletons to be exposed to prenatal risks, including preterm birth where ~ 60% of twin births take place before 37 weeks of gestation as compared to ~ 10% in singletons (Chauhan et al., 2010). In our sample, no participants were extremely preterm (< 28 week of gestation), 7% were very preterm (28 to 32 weeks), 37% categorized as moderate to late preterm (32 to 37 weeks), and 50% were born in gestational week 37 our later. For 6% of the participants, information on gestational age was not available.

Recruitment and data collection in RATSS was conducted between 2011 and 2023. The main recruitment sources were population-based twin studies: the Child and Adolescent Twin Study in Sweden (CATSS) (Anckarsäter et al., 2011) and the Young Adult Twins in Sweden Study (YATSS) (Zagai et al., 2019). Twin-pairs that were likely discordant or concordant for autism or ADHD (according to screening measures in the population-based studies) were prioritized, whereas twin-pairs were neither twin had indications of any neurodevelopmental condition were recruited as controls. Potential participants or their parents were contacted via mail send-outs and those interested in participation received further study information via telephone and in writing. Twin-pair zygosity was determined using a panel of 48 single nucleotide polymorphisms (Hannelius et al., 2007), or by parent-report on a zygosity questionnaire (for 6 pairs).

Twin-pairs where camouflaging and QoL data were not available for one of the twins (24 pairs) or where the twins were of different sex (2 pairs) were excluded. In addition, pairs where one or both twins had an intellectual disability (5 pairs) were excluded, as neither the English nor Swedish version of the CAT-Q has been validated for this population (Hull et al., 2018; Lundin Remnélius & Bölte, 2023), and participants in RATSS with intellectual disability found items challenging in our experience. Finally, twins who did not have zygosity determined at the time of analysis (2 pairs) were also excluded, resulting in the final sample (*N* = 140). Written informed consent was obtained from all participants and/or their caregivers, depending on age.

### Socioeconomic Background

Parent-reported monthly household income before tax is presented (in Swedish crowns, 10 Swedish crowns ≈ 0.85 Euro [€]). Parents’ incomes were distributed in the following ranges, below 20,000 (4%), between 20,000 and 40,000 (14%), between 40,000 and 60,000 (39%), between 60,000 and 80,000 (20%), and more than 80,000 (16%). For 7% of participants no report on household income was available. Regarding parental education, the highest level of education completed by mothers are reported. Of these, 1% reported having finished Swedish primary and lower secondary education, 51% had completed upper secondary education, and 43% had completed a university education (minimum three years). Maternal education was not reported for the remaining 4% of participants. For 61 of the total 70 pairs, one or both parents also reported on their own ethnic background where the majority (95%) reported being of European background.

### Measures

Comprehensive neurodevelopmental and psychiatric assessments were conducted by experienced clinicians during a three-day visit at our clinical research unit, providing information for DSM-5 consensus research diagnoses. Diagnosis of autism was supported by clinician-ratings on the Autism Diagnostic Observation Schedule Generic or 2nd edition (ADOS-G or ADOS-2) (Gotham et al., 2007; Lord et al., 2012) and parent interview on the ADI-R (Rutter et al., 2003). Autistic traits were measured by parent-report on the child or adult version of the Social Responsiveness Scale Second Edition (SRS-2), using total raw scores as is recommended for research purposes (Constantino & Gruber, 2012). Other neurodevelopmental and psychiatric diagnoses, including ADHD, were determined based on multiple information sources including medical history, parent interview on the Kiddie Schedule for Affective Disorders and Schizophrenia (K-SADS) (Kaufman et al., 1997) for participants younger than 18 years, or interview on the Structured Clinical Interview for DSM-IV-Axis I Disorders (SCID-I) for twins aged 18 years or more, in combination with separate twin and parent interviews on the Diagnostic Interview for ADHD in adults (DIVA) (Kooij, 2010). Assessments in RATSS also included IQ testing using the Wechsler Intelligence Scales for Children – Fourth edition (WISC-IV) or the Wechsler Adult Intelligence Scale – Fourth Edition (WAIS-IV) (Wechsler, 2003; Wechsler et al., 2008). Twin sex was based on assigned-at-birth sex (note that the term sex/gender is used in the current study when discussing differences in behavior, as biological and social/experiential influences on behavior are not easily disentangled) (Lai et al., 2015). For the majority of the participants (63 pairs), self-reports on camouflaging and QoL (using the Swedish version of the Camouflaging Autistic Traits Questionnaire (CAT-Q/SE) and EUROHIS-QOL) were collected after the twins’ initial visit in RATSS, with a maximum of eleven years later (*Mdn* = 3 years). The remaining participants (7 pairs) completed all assessments, including CAT-Q/SE and EUROHIS-QOL, at the time of their visit in RATSS.

### CAT-Q/SE

The CAT-Q is a self-report questionnaire encompassing 25 items describing camouflaging behaviors derived from experiences of autistic adults, which are rated on a 7-point Likert scale ranging from *strongly disagree* to *strongly agree*. Items describe experiences such as “in social situations, I feel like I’m ‘performing’ rather than being myself”, and “I have tried to improve my understanding of social skills by watching other people”. All items are summed to a total score ranging between 25 and 175 with higher scores indicating higher levels of camouflaging. The questionnaire was developed with the aim of measuring camouflaging in autistic and non-autistic populations, and its original English version has shown satisfactory psychometric properties (Hull et al., 2018). The Swedish version (CAT-Q/SE) has demonstrated good-to-excellent reliability (Cronbach’s α = 0.93; test-retest stability: *ICC* = 0.85) in autistic and non-autistic samples aged 10 years and older, and support for diagnostic and concurrent validity (Lundin Remnélius & Bölte, 2023).

### EUROHIS-QOL 8-Item Index

QoL was measured using self-report on the EUROHIS-QOL 8-item index (Power et al., 2003), a shortened version of the WHO scale the WHOQOL-BREF. The WHOQOL-BREF has been validated for adolescents from the age of 12 years (Chen et al., 2006), and is probably the most frequently used QoL-measure in autism research (Mason et al., 2018). The EUROHIS-QOL items cover subjective QoL in four domains: psychological, social, physical, and environmental, which are rated on a 5-point Likert scale. Items are summed to create a total score ranging from 8 to 40 with higher scores indicating higher QoL. The scale has shown satisfactory reliability (α = 0.83) and validity across a range of countries (da Rocha et al., 2012; Schmidt et al., 2006).

### Missing Data

One participant had missing data for a single item on the CAT-Q/SE, and the median of the responses on the remaining items was therefore imputed. Thirteen participants had a maximum of three missing items on the SRS-2, and for these items data was imputed in accordance with the SRS-2 manual (Constantino & Gruber, 2012).

### Statistical Analyses

All analyses were conducted in R software, and *p*-values below 0.05 were considered significant. Regression models were fitted using the generalized estimating equations (GEE) framework with doubly robust standard errors (drgee package) (Zetterqvist & Sjölander, 2015), which do not assume normally distributed data. The GEE is a recommended analytic approach in co-twin control designs (Scurrah & Hopper, 2019), and has been used by our group in previous publications (e.g., Isaksson et al., 2019; Lundin Remnélius et al., 2021b; Neufeld et al., 2021). In the current study, we report unstandardized regression estimates, displaying the change in outcome (EUROHIS-QOL total score) associated with a one-point increase on the exposure (CAT-Q/SE total score), and explicitly state when standardized estimates are provided. Regressions were first conducted across the sample and subsequently within twin pairs, as detailed below.

*Across-individuals analyses* are multiple linear regression models utilizing cluster-robust standard errors, accounting for the fact that twins form “clusters” rather than being independent data points. To describe potential group-level differences in camouflaging between autistic and non-autistic participants, a regression model testing the association between autism and camouflaging was fitted which included the covariates age and ADHD as well as the interaction term autism by sex to investigate potential moderation by sex. Subsequently, connected to the second aim, we conducted across-individuals regressions to investigate if an association was found between camouflaging and QoL across the sample. First, an unadjusted model was fitted with camouflaging as the exposure and QoL as the outcome, and secondly, a regression model adjusting for sex, age, autistic traits (SRS-2 total raw scores), and ADHD was fitted.

*Within-pair analyses* were conducted to address the third aim, testing whether the association between camouflaging and QoL can be found also within twin-pairs. These analyses implicitly control for everything shared by the twins within a pair (including shared environment and on average 50% of segregating genes in DZ-pairs and 100% of genes in MZ-pairs). The within-pair analyses evaluate whether the twin who displays increased camouflaging also displays lower QoL, compared to their co-twin. Unadjusted regressions were first fitted, followed by regressions adjusted for autistic traits and ADHD (sex and age are constant within the twin-pairs as both twins are of the same sex and age, and thus already implicitly adjusted for).

In the within-pair analyses, twins that are discordant on exposure (here, CAT-Q/SE total score), covariates, and/or outcome (EUROHIS-QOL total score) are informative for the analyses. In the current sample, 40 MZ pairs showed at least a 1-point discordance for CAT-Q/SE total score, while 28 MZ pairs showed a discordance of at least 8 points, reflecting the standard error of measurement of the CAT-Q/SE^1^. Of the DZ twin-pairs, all 28 pairs displayed at least a 1-point discordancy on CAT-Q/SE, and 21 pairs a discordancy of 8 points or more.

The within-pair models allow testing whether the exposure is associated with the outcome, when holding familial factors constant. These analyses control for shared environment, i.e., environmental factors that make twins similar, including a range of pre- and perinatal factors (e.g., parental age, gestational age), family socioeconomic status, parenting styles, and amount of conflict in the family (Knopik et al., 2017), as well as shared genetics (on average 50% of segregating genes within DZ-pairs and 100% of genetics in MZ-pairs). The within-pair analyses were split by zygosity to allow comparison between the degree of genetics controlled for, where the within-pair association in MZ-pairs is considered the most stringent test of causal effects provided by the co-twin control design (McGue et al., 2010). Thus, if the association remains within both DZ and MZ pairs, the results are consistent with camouflaging having a causal effect on QoL. If the association remains within DZ-pairs but not within MZ-pairs, the results are suggestive of genetic confounding on the relationship (as controlling fully for genetics attenuates the association). The latter scenario could be indicative of genetic pleiotropy, where the same genes influence both the exposure and outcome trait. To assess if the within-pair associations were significantly different in the DZ- and MZ-groups, Wald-type Chi-square tests were run.

Finally, to test if the association between camouflaging and QoL was confounded by internalizing conditions (e.g., anxiety, depression, dysthymia), the adjusted models (across individuals and within-pair) were rerun adjusting also for diagnosis of one or more internalizing conditions at the time of visit in RATSS, coded as a categorical variable.

## Results

### Across Individuals

Autism diagnosis was associated with increased camouflaging (*b* = 39.79 [95% *CI*, 26.38 to 53.19], *p* < .001). Age and sex was not associated with camouflaging, but a significant autism by sex interaction effect suggested that the association between autism and camouflaging was driven by the autistic females in the sample (*b* = − 42.42 [95% *CI*, − 59.48 to − 25.37], *p* < .001), who had higher CAT-Q/SE total scores (*M* = 106.2) than autistic males (*M* = 61.6), as well as non-autistic females (*M* = 60.5) and males (*M* = 60.7). ADHD was also associated with increased camouflaging in the model (*b* = 15.69 [95% *CI*, 7.17 to 24.20], *p* < .001).

Across the sample, camouflaging was associated with decreased QoL in the unadjusted model (*b* = -0.12 [95% *CI*, − 0.16 to − 0.08], *p* < .001). The association was not attenuated when controlling for sex, age, autistic traits, and ADHD in the adjusted model (*b* = -0.12 [95% *CI*, − 0.17 to − 0.07], *p* < .001). Of the covariates, only age showed a significant association with QoL (*b* = − 0.15 [95% *CI* − 0.28 to − 0.01], *p* = .029). The adjusted standardized estimate showed that 1 *SD* increase in camouflaging scores was associated with an average decrease of 0.46 *SD* in QoL score.

### Within-Pairs

**Fig. 1:**
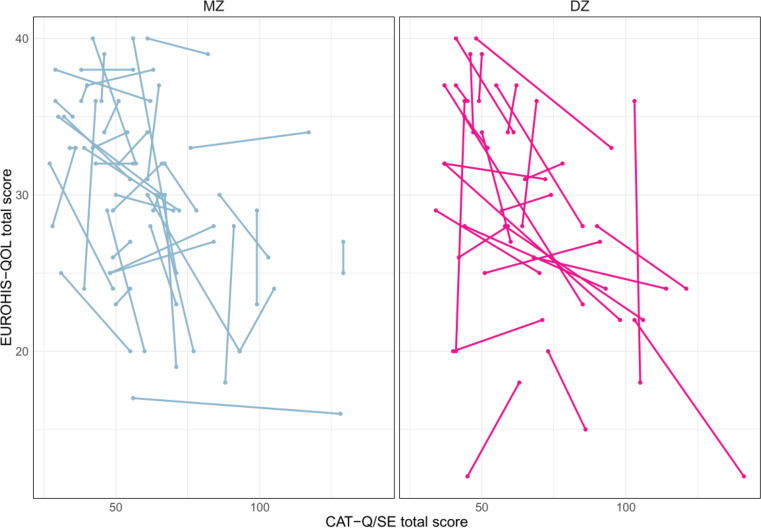
Within-pair associations between camouflaging and QoL in MZ- and DZ-pairs. The twins in a pair are shown connected with a line

In the unadjusted model which implicitly control for unmeasured familial confounders, the twins self-reporting more camouflaging had on average lower QoL than their co-twins within DZ-pairs (*b* = − 0.12 [95% *CI* − 0.17 to − 0.07], *p* < .001), and within MZ-pairs (*b* = − 0.07 [95% *CI* − 0.14 to − 0.01], *p* = .023) (See Fig. 1). Controlling for autistic traits and ADHD in the adjusted models did not attenuate the association within DZ-pairs (*b* = − 0.13 [95% *CI* − 0.21 to − 0.06], *p* < .001) or MZ-pairs (*b* = − 0.07 [95% *CI* − 0.14 to − 0.01], *p* = .016). The within-pair associations did not differ between DZ- and MZ-groups (unadjusted: *χ2* = 1.23, *p* = .267; Adjusted: *χ2* = 1.65, *p* = .199).

### Internalizing Conditions

To account for the influence of previous psychiatric diagnoses on the association between camouflaging and QoL, internalizing conditions were included as a categorical covariate in the adjusted models, across individuals and within-pairs. Overall, this did not change the results, where camouflaging displayed a similar link to QoL both across the sample (*b* = − 0.11 [95% *CI* − 0.16 to − 0.07], *p* < .001), as well as within DZ-pairs (*b* = − 0.12 [95% *CI* − 0.19 to − 0.05], *p* < .001) and MZ-pairs (*b* = − 0.07 [95% *CI* − 0.13 to − 0.01], *p* = .028). In the within-pair model, having a previous internalizing condition was negatively associated with QoL (*b* = − 2.46 [95% *CI* − 4.66 to − 0.27], *p* = .028).

## Discussion

In the current study, a co-twin control design was utilized to investigate the potential causal relationship between camouflaging and QoL in a sample of non-autistic and autistic twins, providing control for unmeasured confounding not accounted for in previous research. Autistic participants self-reported higher levels of camouflaging than non-autistic participants, which was driven by the autistic females. Across the sample, a negative association between camouflaging and QoL was found, beyond the influence of autistic traits, ADHD, sex, and age. In line with our hypotheses, the association between camouflaging and QoL remained when controlling for shared environment and genetics within DZ- and MZ-pairs, implying that the link cannot be explained by familial confounding. Thus, the within-pair results may reflect a causal influence of camouflaging on QoL.

Autistic participants self-reported higher levels of camouflaging than non-autistic participants, suggesting that while non-autistic people might have similar experiences, masking and compensating for autistic traits is generally more extensive among people on the autism spectrum, consistent with findings in previous research (Hull et al., 2018; Jedrzejewska & Dewey, 2022). In the current study, the difference was driven by autistic females self-reporting elevated camouflaging compared to the other groups. Our results are thus in line with previous reports suggesting that autistic girls and women on group level display a higher propensity to camouflage than autistic boys and men (Cola et al., 2020; Hull et al., 2019; Lai et al., 2017). This difference could be driven by both individual-level and contextual factors. Potential contributors include increased social motivation to interact and form relationships among autistic females compared to autistic males (Head et al., 2014; Sedgewick et al., 2016), along with differing social environments where girls may be expected to show more interpersonal competence and sensitivity to social nuances (Kreiser & White, 2014). Still, the sex/gender moderation in our data should be interpreted with necessary caution as the groups of autistic females (*n* = 8) and males (*n* = 14) were small, and while age was added as a covariate in the model, autistic females were substantially older than autistic males. In the same model, ADHD was linked to increased camouflaging of autistic traits, in line with emerging evidence (van Der Putten et al., 2024). People with ADHD display elevated autistic traits (Bölte et al., 2018), which might contribute to the finding of elevated camouflaging observed in our data. On the other hand, increased CAT-Q/SE scores in ADHD may also indicate that the questionnaire picks up on behaviors that are related to neurodivergence rather than specifically autistic traits, as has been suggested (Fombonne, 2020; Lundin Remnélius & Bölte, 2023). Future studies should further investigate camouflaging levels in different clinical populations, including people with ADHD, to assess to what extent similar social coping strategies are used across diagnostic groups.

A negative association between camouflaging and QoL was observed across the sample, consistent with previous reports (Milner et al., 2022; Lundin Remnélius & Bölte, 2023). Also, in accordance with our hypothesis, the association remained within both DZ- and MZ-pairs meaning that the link persists even when accounting for a range of alternative explanations for the association including shared environmental or genetic confounding. Speculatively, camouflaging might have a direct effect on QoL and/or indirect effects mediated by symptoms of depression and perceived stress, which have shown correlations with camouflaging (Cage & Troxell-Whitman, 2019; Hull et al., 2018). Similarly, the link between camouflaging and suicidality has been suggested to be mediated by experiences of being alone and disconnected from the social surroundings (Cassidy et al., 2020), and similar experiences might play a role in mediating the relationship between camouflaging and QoL. In summary, our results add to the body of data suggesting that camouflaging might be a risk factor for unfavorable outcomes including low QoL, in line with qualitative reports from autistic people (Hull et al., 2017), and subsequently also from people without an autism diagnosis (Bernardin et al., 2021b; Livingston et al., 2019). By ruling out several alternative non-causal explanations for the association, our results allow for a stronger causal claim than previous cross-sectional studies.

Identification of risk factors that have negative causal effects on QoL is an essential step in developing methods for prevention and interventions that aim to support autistic and non-autistic people in promoting satisfaction in areas of life seen as important by the individuals themselves. Our findings emphasize the relevance of assessing camouflaging in mental health services and that people displaying extensive camouflaging should be followed-up regarding potential negative effects including reductions in QoL. As a range of studies have found camouflaging to be elevated among autistic people and particularly among autistic girls/women, assessment of such strategies is warranted in these populations given the proposed risks of detrimental effects. It should however be noted that some autistic individuals also perceive camouflaging to have positive effects on participation, such as facilitating in getting a job or developing friendships (Hull et al., 2017), suggesting that any support should be carefully adapted to the individual, e.g., encouraging discussion and reflection regarding use of different camouflaging strategies and their potential impact on mental health (Hull et al., 2021). A balanced use of camouflaging strategies may be preferential to facilitate functioning and mental health, allowing the individual to gain potential beneficial effects (e.g., in building social relationships) while minimizing detrimental impact on feelings of authenticity and QoL. Importantly, as autistic people commonly experience external pressures to camouflage, and might mask autistic traits in order to avoid stigma and bullying, interventions likely also have to target environmental factors in order to be effective (Bölte et al., 2019; Mandy, 2019). Previous findings suggest that autistic people engage in less camouflaging in interactions with accepting and supportive social partners (Cook et al., 2023), implying that environments should adopt a more tolerant approach allowing less camouflaging. Such an approach may be crucial to avoid potential barriers for effective health care provision for autistic people (Mason et al., 2021). Given that autism knowledge among neurotypical raters is associated with more positive judgements of autistic people (Sasson & Morrison, 2019), interventions aiming to increase autism knowledge and tolerance among non-autistic people may reduce the need to camouflage autistic traits.

The within-pair estimate was descriptively slightly lower in MZ-pairs compared to DZ-pairs, although not significantly different. It should be noted that within-pair associations, particularly within MZ-pairs, are susceptive to measurement error which can attenuate the association (McGue et al., 2010). Nevertheless, our study is the first to observe a link between camouflaging and QoL while ruling out a number of alternative scenarios including that the association is due to familial confounding. Our results thus warrant further research, including investigation of the link between camouflaging and QoL as well as mental health problems in larger twin-samples, and longitudinal research of camouflaging and negative outcomes.

### Limitations and Strengths

Using the operationalization of the CAT-Q, the current study measured only certain aspects of camouflaging. The CAT-Q captures camouflaging intention and experiences but will not pick up on whether camouflaging attempts are “successful” or not (Hull et al., 2018). A person who attempts and fails at camouflaging could suggest a strong social motivation to interact and form relationships but challenges in achieving this, which could have a detrimental effect on QoL (Lai & Baron-Cohen, 2015; Lundin Remnélius et al., 2021a). On the other hand, “successful” camouflaging might also have a negative effect on QoL if challenges are not acknowledged by others, so that the social demands are persistently exceeding the individual’s capacity. Further research into hypothetical detrimental effects of camouflaging should include measures from different informants, such as parent-or-other-report on camouflaging “success”. Interestingly, a recent study found that camouflaging operationalized as the discrepancy between self-reported autistic traits and clinician-rated autism characteristics (likely capturing “successful” camouflaging) was strongly associated with the total score on a parent-report CAT-Q version, while the correlation with self-reported camouflaging was small (Hannon et al., 2022), indicating that the different measures capture overlapping but different aspects of camouflaging.

Both main variables in the current study were assessed using self-report questionnaires, which might have introduced bias in the measurements. Still, the CAT-Q captures camouflaging intentions which might not be observable by others. Similarly, subjective appraisal is at the heart of the QoL construct, which was measured using a well-established scale developed by the WHOQOL group (da Rocha et al., 2012; Jonsson et al., 2017; World Health Organization, 1998). Also, self- and proxy-reports are not interchangeable for either camouflaging or QoL, emphasizing the importance of the first-person perspective in measures of these constructs (Hannon et al., 2022; Knuppel et al., 2018; Moss et al., 2017). In autism research, a range of studies have investigated “objective” QoL outcomes, including educational level, employment, and living situation. Yet, these outcomes do not sufficiently predict how an individual perceives their QoL (Helles et al., 2017), emphasizing the need for subjective report. Also, previous studies support that autistic people can provide reliable first-hand reports of QoL (Hong et al., 2016; McConachie et al., 2018; Williams et al., 2022). Nevertheless, future research should investigate the link between camouflaging and outcomes assessed by objective measures in addition to self-reports, e.g., in education and employment, and biomarkers of stress or anxiety. It should also be acknowledged that the self-report questionnaires used in our study required that twins with intellectual disability in the RATSS sample were excluded, limiting the generalizability of our findings to autistic and non-autistic people without intellectual disability.

The current study encompassed a wide age range among the participants. While the analyses were adjusted for age ensuring that this variable did not drive the results, there is still a possibility that the link between camouflaging and QoL was mainly driven by a particular age group. Although our sample size did not allow for further stratification on age, future studies should assess camouflaging outcomes in subgroups with narrower age ranges, as it has been shown that camouflaging levels vary during the lifetime (Lundin Remnélius & Bölte, 2023).

Furthermore, the Swedish version of the CAT-Q has been validated in autistic and non-autistic samples displaying good-to-excellent reliability. However, some issues regarding construct validity were observed, and particularly in participants younger than 15 years were the link between autism and CAT-Q/SE scores appeared to be weaker than among older participants (Lundin Remnélius & Bölte, 2023). The current study included twins younger than 15 years, which should be considered when interpreting the results, and warrants future replication of our results in samples with a higher minimum age. In addition, the three-factor structure of the original CAT-Q could not be replicated in the Swedish validation, suggesting that further investigation of the psychometric properties of the CAT-Q/SE should be conducted.

Although the use of QoL as an outcome measure allows people to appraise their own position in areas that are meaningful for the individual, a discussion regarding the utility of general population QoL-scales in autistic populations has emerged. One study found that the WHOQOL-BREF scale, which the QoL-scale in the current study is based on, captures relevant aspects of QoL according to autistic adults but may miss some areas of specific importance to autistic people, e.g., the level of knowledge about autism among others (McConachie et al., 2020). Consistently, researchers have called for development of QoL measures that are tailored for people on the autism spectrum (Moss et al., 2017). Thus, the measure used in our study might have omitted important areas of QoL among the autistic participants.

For the majority of participants, data on autistic traits were collected before the twins provided self-reports on camouflaging and QoL and might not provide precise measures of current levels of autistic characteristics. The minority of participants who completed all assessments at the same timepoint were too few (7 twin-pairs) to conduct meaningful separate analyses in this subsample. Still, autistic traits are found to be reasonably stable over time, also when time points of measurements are several years apart (Constantino et al., 2009; Haraguchi et al., 2019; Robinson et al., 2011). Similarly, assessments of internalizing psychiatric conditions were made prior to the collection of camouflaging and QoL data. These conditions were included in the current study as a covariate to provide adjustment for confounding by prior psychiatric conditions, which could influence both camouflaging and QoL. Internalizing symptoms and conditions show reasonable stability over time, especially from adolescence onwards (Carballo et al., 2010; Nivard et al., 2015). Nevertheless, our findings of the association between camouflaging and overall QoL withstanding influence from internalizing conditions warrants replication. Further insights into the direction of the relationship between internalizing problems and camouflaging are needed.

The co-twin control design allows for ruling out alternative scenarios other than a causal relationship but is not in itself sufficient for establishing a causal effect, due to a number of limitations. First, while an association within MZ-pairs is consistent with a causal effect, the within-pair analyses cannot rule out that other non-shared environmental factors, e.g., experiences affecting one twin but not the other, could influence both exposure and outcome. Second, the design does not rule out the possibility of reversed causation, where lower QoL contributes to increased camouflaging, or a potential bidirectional relationship. Therefore, future longitudinal research is required to disentangle the direction of the relationship. Third, as the within-pair analyses require splitting the sample by zygosity and depend on twin-pairs that are discordant on exposure and/or outcome these analyses have reduced statistical power compared to analyses conducted across individuals (D’Onofrio et al., 2020). Power calculations are complicated in the current study due to the sample not being randomly selected and challenges in translating findings in singletons to expected effects within twin-pairs. In our model, each strata (i.e., twin pair) has its own “sub-model” in which it could theoretically be possible to calculate standardized regression coefficients and explained variance, but this would differ between all strata and therefore not be useful. While our sample was likely underpowered to detect small effects, the co-twin control design has been used by our and other groups in samples of comparable size to discover medium-to-large effects (e.g., Cauvet et al., 2019; Lundin Remnélius et al., 2021b; Wilson et al., 2015). In addition, the RATSS sample is thoroughly phenotyped through an assessment procedure spanning three days, giving a depth of data not easily replicated in larger twin samples. Therefore, findings in the RATSS twin-sample can be seen as an important complement to findings in larger twin samples, and vice versa. Finally, the co-twin control design assumes that effects observed in twins can be generalized to non-twin populations.

## Conclusions

The current study investigated the association between camouflaging and QoL, utilizing a co-twin control design controlling for unmeasured familial confounding. The results indicated a negative association between camouflaging and QoL, beyond the influence of sex/gender, age, ADHD diagnosis, and autistic traits. The association remained within DZ- and MZ-pairs, suggesting a link beyond familial confounding, which is consistent with a causal effect of camouflaging on QoL. The results show that the association survives adjustment for a multitude of alternative confounding scenarios and are thus in line with previous qualitative reports of camouflaging having negative effects on QoL. The results are of particular importance to autistic people where the use of such strategies is elevated and QoL is commonly found to be reduced. Our findings warrant that clinicians assess camouflaging in order to prevent potential detrimental consequences. Interventions addressing camouflaging likely need to involve environmental factors such as increasing knowledge and tolerance of neurodiversity and autism in the general public.

## Footnotes

1 Calculated from internal consistency and *SD* reported in Lundin Remnélius and Bölte (2023) using the formula $SD\sqrt{1-{r_x}}$, where $r_x$ is the reliability (here, internal consistency).
